# Transcriptome-Based Co-Expression of BRD4 and PD-1/PD-L1 Predicts Poor Overall Survival in Patients With Acute Myeloid Leukemia

**DOI:** 10.3389/fphar.2020.582955

**Published:** 2021-02-01

**Authors:** Cunte Chen, Ling Xu, Rili Gao, Shunqing Wang, Yuping Zhang, Caixia Wang, Chengwu Zeng, Yangqiu Li

**Affiliations:** ^1^ Institute of Hematology, School of Medicine, Key Laboratory for Regenerative Medicine of Ministry of Education, Jinan University, Guangzhou, China; ^2^ Department of Hematology, First Affiliated Hospital, The Clinical Medicine Postdoctoral Research Station, Jinan University, Guangzhou, China; ^3^ Department of Hematology, Guangzhou First People’s Hospital, School of Medicine, South China University of Technology, Guangzhou, China

**Keywords:** acute myeloid leukemia, BRD4, PD-1, PD-L1, prognosis

## Abstract

Positive response to PD-1/PD-L1 blockades was observed in the treatment of solid tumors. However, the clinical response to PD-1/PD-L1 blockade varied in patients with acute myeloid leukemia (AML). It is thought that there are factors other than PD-1 and PD-L1 that may affect the effect of immunotherapy. This study explored the impact of transcriptome-based co-expression of bromodomain containing 4 (BRD4) and PD-1/PD-L1 on the overall survival (OS) of patients with AML, in order to understand whether BRD4 would affect the effect of PD-1/PD-L1 blockades. Bone marrow samples from 59 AML patients in our clinical center and data of 176 patients from the Cancer Genome Atlas (TCGA) database were used for OS analysis and validation. It was found that increased expression of BRD4 was associated with poor OS in AML patients. Moreover, co-expression of BRD4 with PD-1 or PD-L1 was related to poor OS. The co-expression of BRD4 and PD-L1 was better than BRD4 and PD-1 for OS prediction. Furthermore, co-expression of BRD4 and PD-L1 was positively correlated with high tumor mutation burden, which contributed to poor OS in AML patients. Additionally, the co-expression of BRD4 and PD-L1 was associated with poor OS in non-acute promyelocytic leukemia patients with intermediate/high risk or under 60 years. Our results suggest that transcriptome-based co-expression of BRD4 and PD-L1 is a predictor for poor OS in AML patients, which might provide novel insight into designing combinational targeted therapy for AML.

## Introduction

Immune evasion and abnormal immune surveillance of cancers play a crucial role in carcinogenesis and cancer progression (Dunn et al., 2002). The role of programmed cell death 1 (PD-1) and PD-1 ligand 1 (PD-L1) in the immune escape of cancer cells makes them promising targets of cancer therapy (Ghahremanloo et al., 2019; Akin Telli et al., 2020). Recently, blockades of the PD-1/PD-L1 axis have been proven to be successful in the treatment of solid tumors (Liu, 2019; Liu et al., 2019). Moreover, several clinical trials using anti-PD-1 or PD-L1 antibodies are ongoing to treat patients with acute myeloid leukemia (AML) (Hobo et al., 2018). Previous reports have shown that overexpression of PD-1 and PD-L1 in AML patients is associated with poor clinical outcome (Huang et al., 2019; Chen et al., 2020). Additionally, PD-L1 expression is closely related to the positive response of PD-1/PD-L1 blockade in solid tumor therapy (Balar et al., 2017; Koemans et al., 2019; Yu et al., 2020). These findings suggest that PD-1/PD-L1 blockade may be a novel immunotherapeutic strategy for AML. However, the clinical response to PD-1/PD-L1 blockade varied in different AML patients (Stahl et al., 2019). It is thought that there are factors other than PD-1 and PD-L1 that may aggravate their immunosuppression, influence their effects on immunotherapy, and contribute to the poor prognosis of AML patients (Stahl et al., 2019; Chen et al., 2020).

Bromodomain protein 4 (BRD4) is a member of the bromodomain and extraterminal domain (BET) family that plays pivotal roles in cell cycle and transcription (Devaiah et al., 2016). Abnormal expression of BRD4 is associated with pathogenesis of solid tumors and leukemia. Recently, BRD4 has been identified to be a potential therapeutic target in hematologic malignancies, including AML (Zuber et al., 2011; Fiskus et al., 2014). Interestingly, a BRD4 inhibitor enhanced the efficacy of PD-1 antibody therapy in non-small cell lung cancer patients with Kirsten rat sarcoma 2 viral oncogene homolog (KRAS) mutations (Adeegbe et al., 2018). In addition, PD-L1 is downstream of BRD4, and BRD4 inhibition facilitates the anti-tumor immune response in cancer patients by suppressing PD-L1 expression (Zhu et al., 2016; Hogg et al., 2017). Therefore, it is important to understand the correlation between BRD4 and PD-1/PD-L1 axis in AML patients.

In this study, we investigated the prognostic value of transcriptome-based co-expression of BRD4 and PD-1 or BRD4 and PD-L1 in bone marrow (BM) samples from AML patients in our clinical center. The results were further validated by high-throughput sequencing data from the Cancer Genome Atlas (TCGA) database in a larger number of patients.

## Materials and Methods

### BM Samples

The BM samples were obtained from 59 AML patients at the Guangzhou First People’s Hospital from January 1, 2013 to December 31, 2018. The last follow-up was performed on May 5, 2020, and the median follow-up time for survival was 1,447 days (range: 682–2,176 days). The data of these samples was used as the training cohort. The Overall survival (OS) was defined as the time from the date of diagnosis to the date of death or last follow-up time. Moreover, BM samples from 12 healthy individuals were collected and used as controls. The clinical information of the patients in the training cohort are listed in Table 1. This study was conducted according to the principals of the Declaration of Helsinki and was approved by the Ethics Committee of Guangzhou First People's Hospital. All participants provided their written informed consents.

**TABLE 1:** Clinical characteristics of AML patients in our clinical center.

Variables	Patients (total *n* = 59)
Age, mean ± SD, years	42 ± 16
Gender, *n* (%)	
Female	30 (50.8)
Male	29 (49.2)
Risk stratification, *n* (%)	
Low	13 (22.0)
Intermediate	29 (49.2)
High	7 (11.9)
Unknown	10 (16.9)
Cytogenetic abnormality, *n* (%)	
No	28 (47.5)
Yes	19 (32.2)
Unknown	12 (20.3)
CBF-AML	
No	38 (64.4)
Yes	7 (11.9)
Unknown	14 (23.7)
NPM1 mutation	
No	40 (67.8)
Yes	5 (8.5)
Unknown	14 (23.7)
FLT3 mutation	
No	39 (66.1)
Yes	6 (10.2)
Unknown	14 (23.7)
Subtype, *n* (%)	
M1	1 (1.7)
M2	18 (30.5)
M3	7 (11.9)
M4	5 (8.5)
M5	21 (35.6)
M6	2 (3.4)
Unclassified	5 (8.4)
allo-HSCT, *n* (%)	
Yes	25 (42.4)
No	33 (55.9)
Unknown	1 (1.7)
Follow-up, median (range), days	1,447 (682–2,176)
Status	
Alive	31 (52.5)
Dead	28 (47.5)

SD, standard deviation; CBF-AML, core binding factor acute myeloid leukemia; NPM1, nucleophosmin 1; FLT3, fms related receptor tyrosine kinase 3; allo-HSCT, allogeneic hematopoietic stem cell transplantation.

### TCGA Dataset

The level 3 RNA sequencing and mutation data of 176 *de novo* AML patients was obtained from the TCGA (https://cancergenome.nih.gov/) database by UCSC XENA (https://xenabrowser.net/datapages/) (Chen et al., 2020). These RNA sequencing data from the TCGA database comprised the validation cohort for OS analysis. Tumor mutation burden (TMB) was defined as the number of somatic mutations per mega base (Mb) in the genome. The TMB of each specimen was calculated as the total number of non-synonymous somatic mutations divided by the size of the exons (38 Mb) (Chalmers et al., 2017). The clinical characteristics of the 176 AML patients from the TCGA database were reported in a previous study (Chen et al., 2020). The TCGA dataset was used to validate the results of the training cohort.

### Quantitative Real-Time PCR (qRT-PCR)

Extraction of total RNA and reverse transcription of RNA into cDNA were performed according to the manufacturer’s instructions (Chen et al., 2019). The relative expression levels of BRD4, PD-1, and PD-L1 were detected using a qRT-PCR kit (TIANGEN, China) (Zeng et al., 2018), and 18S rRNA was used as an internal control. The sequences of the primers used for qRT-PCR are listed in Table 2. The results are presented as 2^−ΔΔCT^.

**TABLE 2:** Primers for qRT-PCR.

Target	Sequence 5′–3′
PD-1 (F)	CTC​AGG​GTG​ACA​GAG​AGA​AG
PD-1 (R)	GAC​ACC​AAC​CAC​CAG​GGT​TT
PD-L1 (F)	TAT​GGT​GGT​GCC​GAC​TAC​AA
PD-L1 (R)	TGC​TTG​TCC​AGA​TGA​CTT​CG
BRD4 (F)	AGG​CAA​AAG​GAA​GAG​GAC​G
BRD4 (R)	CGA​TGC​TTG​AGT​TGT​GTT​TGG
18S rRNA (F)	CGG​CGG​CTT​TGG​TGA​CTC​TAG​A
18S rRNA (R)	CCT​GCT​GCC​TTC​CTT​GGA​TGT​G

### Statistical Analysis

**FIGURE 1:**
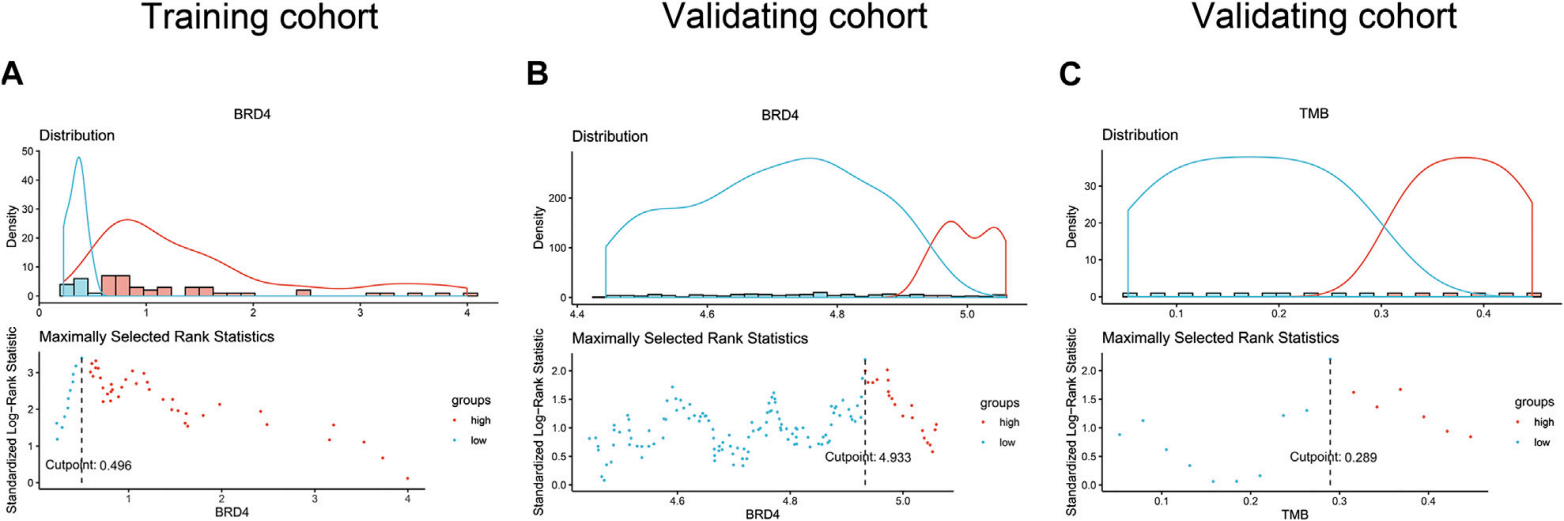
The optimal cutoff values of BRD4 in the training **(A)** and validating **(B)** cohorts, and TMB in the TCGA data **(C)**.

All statistical analyses were performed using Statistical Product and Service Solutions (SPSS) (version 22.0, IBM, Armonk, NY, United States) software and *R* (version 3.6.1, https://www.r-project.org/). The function “surv_cutpoint” in the “survminer” R package determined the optimal cutoff value for continuous variables (Figures 1A–C), that were used to plot Kaplan-Meier curves. A log-rank test was applied to compare difference between groups. COX proportional hazards models were constructed with the “survival” R package. Correlation analysis was shown by Spearman’s coefficient. Comparison of categorical variables was performed by chi-square test. A two-tailed *p* value <0.05 was considered statistically significant.

## Results

### Co-Expression of BRD4 and PD-1 or PD-L1 Is Associated With Poor OS in AML

**FIGURE 2:**
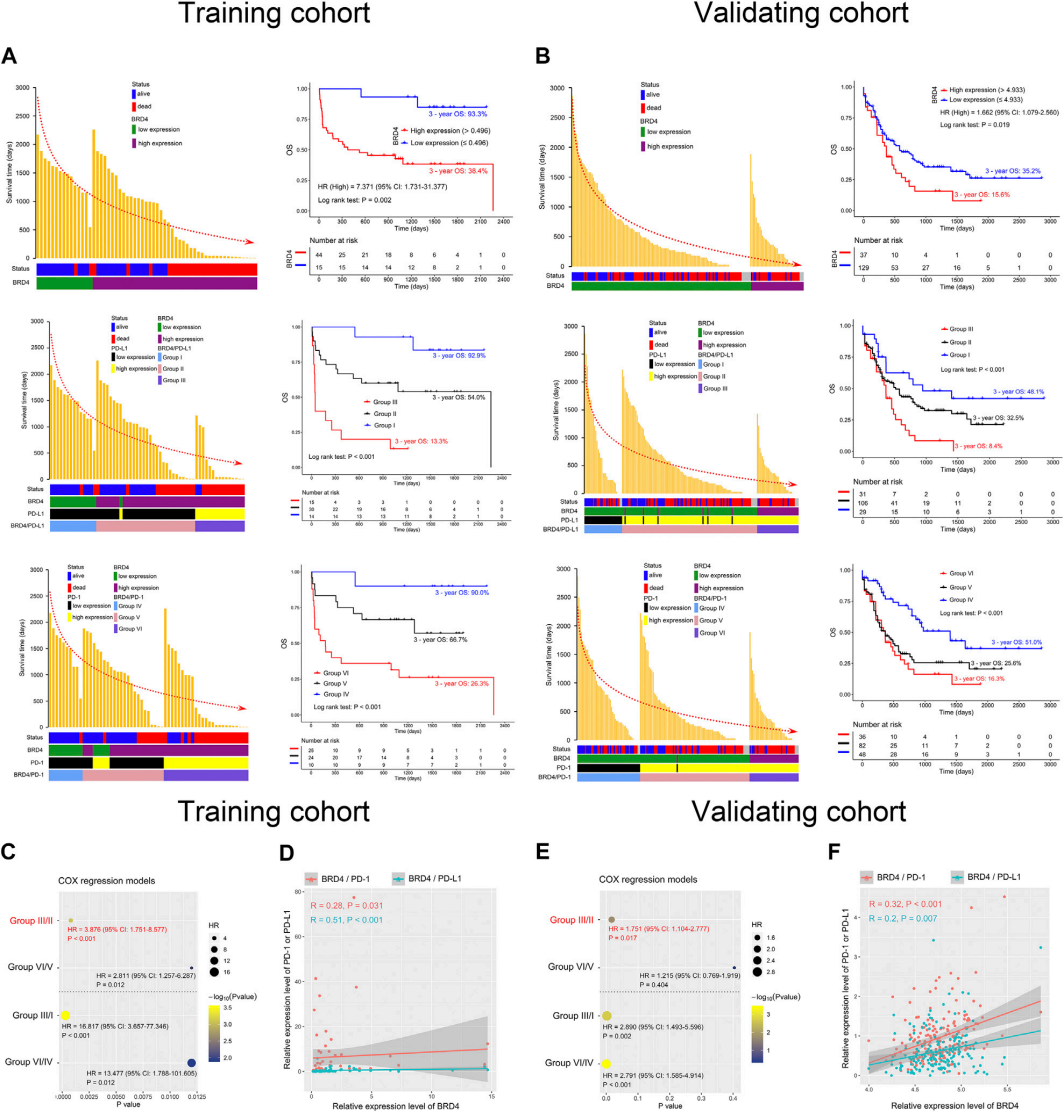
Co-expression of BRD4 and PD-1 or PD-L1 predicts poor overall survival (OS) in AML patients. **(A,B)** Survival time distribution (left panel) and Kaplan-Meier curves (right panel) plotted according to BRD4 expression status (upper panel), and the BRD4/PD-L1 (middle panel) and BRD4/PD-1 (lower panel) combinations in the training **(A)** and validating **(B)** cohorts. The red dotted line represents the change of survival time. **(C,E)** COX proportional hazards models constructed based on BRD4/PD-L1 or BRD4/PD-1 expression in the training **(C)** and validating **(E)** cohorts. **(D,F)** Analysis of the correlation between BRD4 and PD-L1/PD-1 using Spearman’s method for the training **(D)** and validating **(F)** cohorts. Group I: BRD4^low^PD-L1^low^; Group II: BRD4^high^PD-L1^low^ or BRD4^low^PD-L1^high^; Group III: BRD4^high^PD-L1^high^; Group IV: BRD4^low^PD-1^low^; Group V: BRD4^high^PD-1^low^ or BRD4^low^PD-1^high^; Group VI: BRD4^high^PD-1^high^; HR: hazard ratio.

**FIGURE 3:**
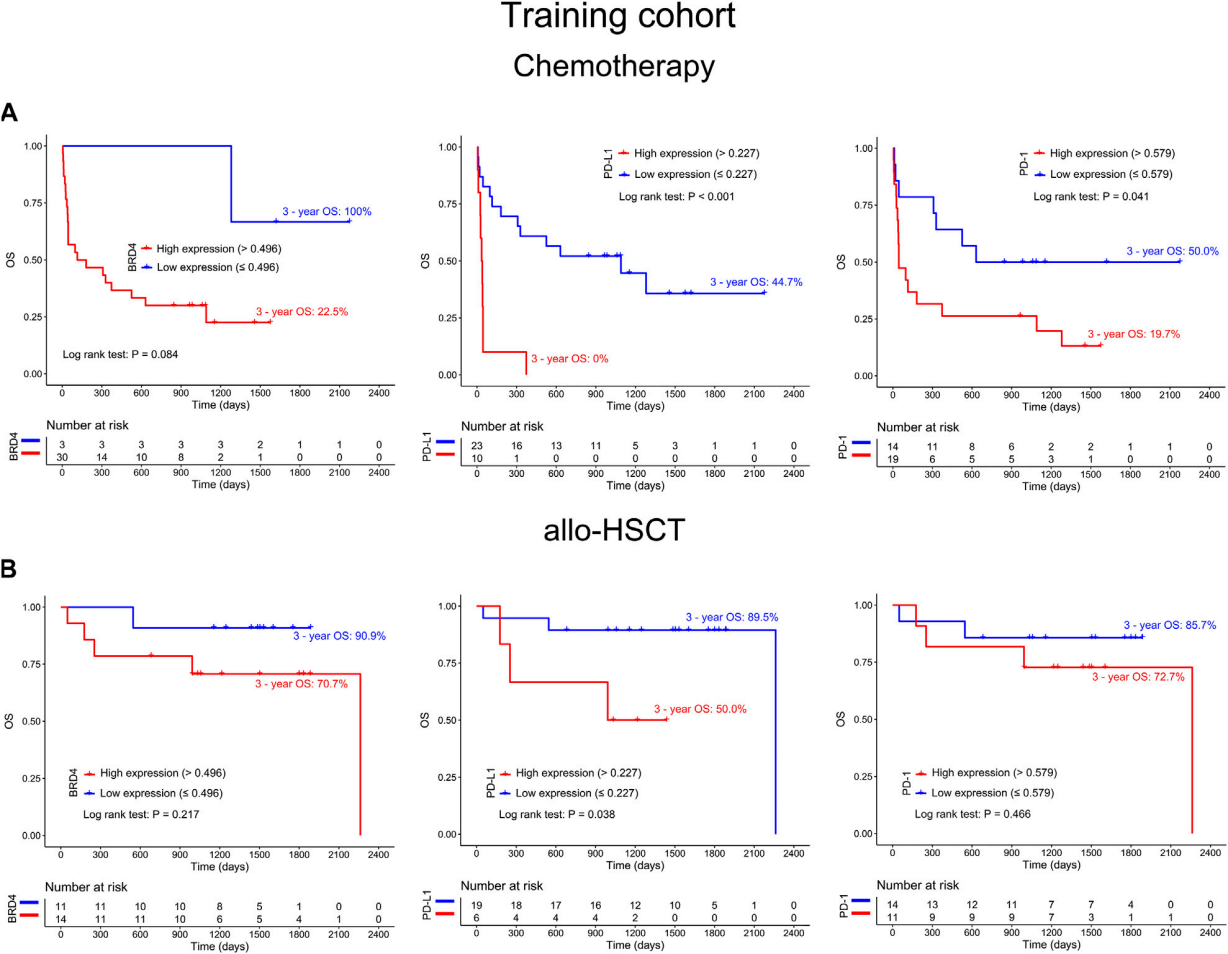
The differences in survival curves between chemotherapy **(A)** and allogeneic hematopoietic stem cell transplantation (allo-HSCT) **(B)** groups according to the expression status of BRD4 (left panel), PD-L1 (middle panel) and PD-1 (right panel) in the training cohort.

To explore the prognostic significance of BRD4 in AML patients, a Kaplan-Meier curve was generated. Higher expression of BRD4 was associated with shorter survival time and poorer OS in the training cohort (3-year OS 38.4% vs. 93.3%, hazard ratio (HR) = 7.371, *p* = 0.002). This finding was confirmed in the validating cohort (3-year OS 15.6% vs. 35.2%, HR = 1.662, *p* = 0.019) (Figures 2A,B, upper panel). To further determine whether allogeneic hematopoietic stem cell transplantation (allo-HSCT) impact on results in terms of gene expression vs outcome, AML patients were divided into chemotherapy only and allo-HSCT groups. The results showed a clear tendency that high expression of BRD4 was related to poor OS in chemotherapy group (*p* = 0.084), while the expression level of BRD4 was not associated with OS in the allo-HSCT group (*p* = 0.217). Similar findings could be observed in PD-1 (*p* = 0.041, *p* = 0.466, respectively). Interestingly, high expression of PD-L1 could predict poor OS in both chemotherapy (*p* < 0.001) and transplantation (*p* = 0.038) groups (Figures 3A,B). These findings indicated that allo-HSCT has no significant impact on the relationship between gene expression and outcome. In addition, core binding factor acute myeloid leukemia (CBF-AML), nucleophosmin 1 (NPM1) and fms related receptor tyrosine kinase 3 (FLT3) in risk stratification have no impact on the expression of BRD4, PD-L1 and PD-1 (Supplementary Figures S1A–C).

Considering that PD-L1 is downstream of BRD4 and BRD4 has a relationship with PD-1, we characterize the prognostic value of co-expression of BRD4 and PD-1 or BRD4 and PD-L1 in AML. Based on Spearman’s correlation analysis, we found that the expression of BRD4 was positively correlated with the expression of PD-L1 (*R* = 0.51, *p* < 0.001) and PD-1 (*R* = 0.28, *p* = 0.031) in the training cohort. This co-expression pattern was confirmed in the validating cohort (*R* = 0.2, *p* = 0.007; *R* = 0.32, *p* < 0.001) (Figures 2D,F). Based on the expression levels of BRD4 and PD-L1, AML patients were divided into three groups: group I, BRD4^low^PD-L1^low^; group II, BRD4^high^PD-L1^low^ or BRD4^low^PD-L1^high^; and group III, BRD4^high^PD-L1^high^. Similarly, based on the expression levels of BRD4 and PD-1, patients were divided into another three groups: group IV, BRD4^low^PD-1^low^; group V, BRD4^high^PD-1^low^ or BRD4^low^PD-1^high^; and group VI, BRD4^high^PD-1^high^. Using the co-expression of BRD4 and PD-L1 to evaluate the OS, lower OS was observed in group III compared to groups II and I in both the training and validating cohorts (3 years 13.3% vs. 54% and 92.9%, and 8.4% vs. 32.5% and 48.1%, respectively) (*p* < 0.001, Figures 2A,B, middle panel). Similar findings were observed in group VI compared to groups V and IV (3 years 26.3% vs. 66.7% and 90%, and 16.3% vs. 25.6% and 51%, respectively) when the co-expression of BRD4 and PD-1 were used to predict OS in AML (*p* < 0.001, Figures 2A,B, lower panel). To better understand the co-expression of BRD4 and PD-L1or PD-1 in predicting OS in patients with AML, Cox regression was used to determine the optimal combination. We found that co-expression of BRD4 and PD-L1 was better than BRD4 and PD-1 [HR (group III/II) vs. HR (group VI/V) 3.876 vs. 2.811, *p* < 0.001 vs. *p* = 0.012] in predicting the OS of the training cohort. This result was confirmed in the validating cohort [HR (group III/II) vs. HR (group VI/V) 1.751 vs. 1.215, *p* = 0.017 vs. *p* = 0.404] (Figures 2C,E).

### Co-Expression of BRD4 and PD-L1 Was Positively Correlated With High TMB

**FIGURE 4:**
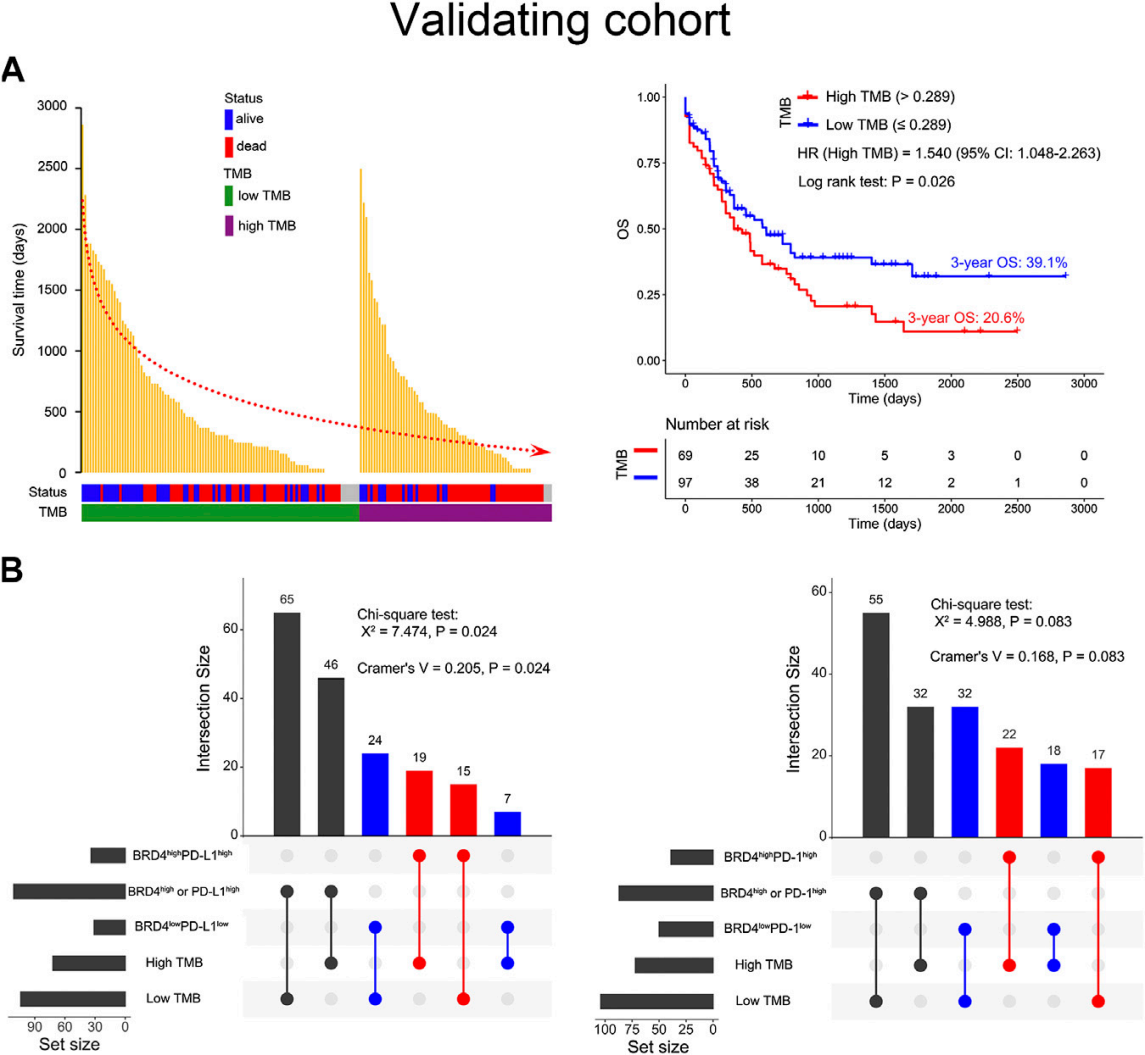
Relationship between BRD4, the PD-L1 or PD-1 combination, and tumor mutation burden (TMB) in the validating cohort. **(A)** The survival time distribution (left panel) and survival curves (right panel) depicted based on the TMB status of the AML patients. The red dotted line represents the trend in survival time with the change in TMB status. **(B)** The Chi-square test used to evaluate the correlation between BRD4 and PD-L1 or PD-1 co-expression and TMB status. The Cramer’s V value ranged from −1 to 1 where the higher values represent a stronger correlation. The connections indicate an intersection of two variables.

To our knowledge, high TMB is closely associated with a positive response to immune checkpoint blockade (ICB). Thus, a total of 176 AML patients from the TCGA database were used to explore the relationship among BRD4, PD-L1, PD-1, and TMB. It was found that higher TMB predicted shorter survival time and lower OS (3-year OS 20.6% vs. 39.1%, HR = 1.540, *p* = 0.026) in AML patients (Figure 4A). Furthermore, chi-square test analysis suggested that co-expression of BRD4 and PD-L1 was positively correlated with high TMB (X^2^ = 7.474, Cramer’s V = 0.205, *p* = 0.024), and there was a clear tendency that co-expression of BRD4 and PD-1 was associated with high TMB (X^2^ = 4.988, *p* = 0.083) (Figure 4B).

### Co-Expression of BRD4 and PD-L1 Is Associated With Poor OS in Non-APL Patients With Intermediate/High Risk or Under 60 Years Old

**FIGURE 5:**
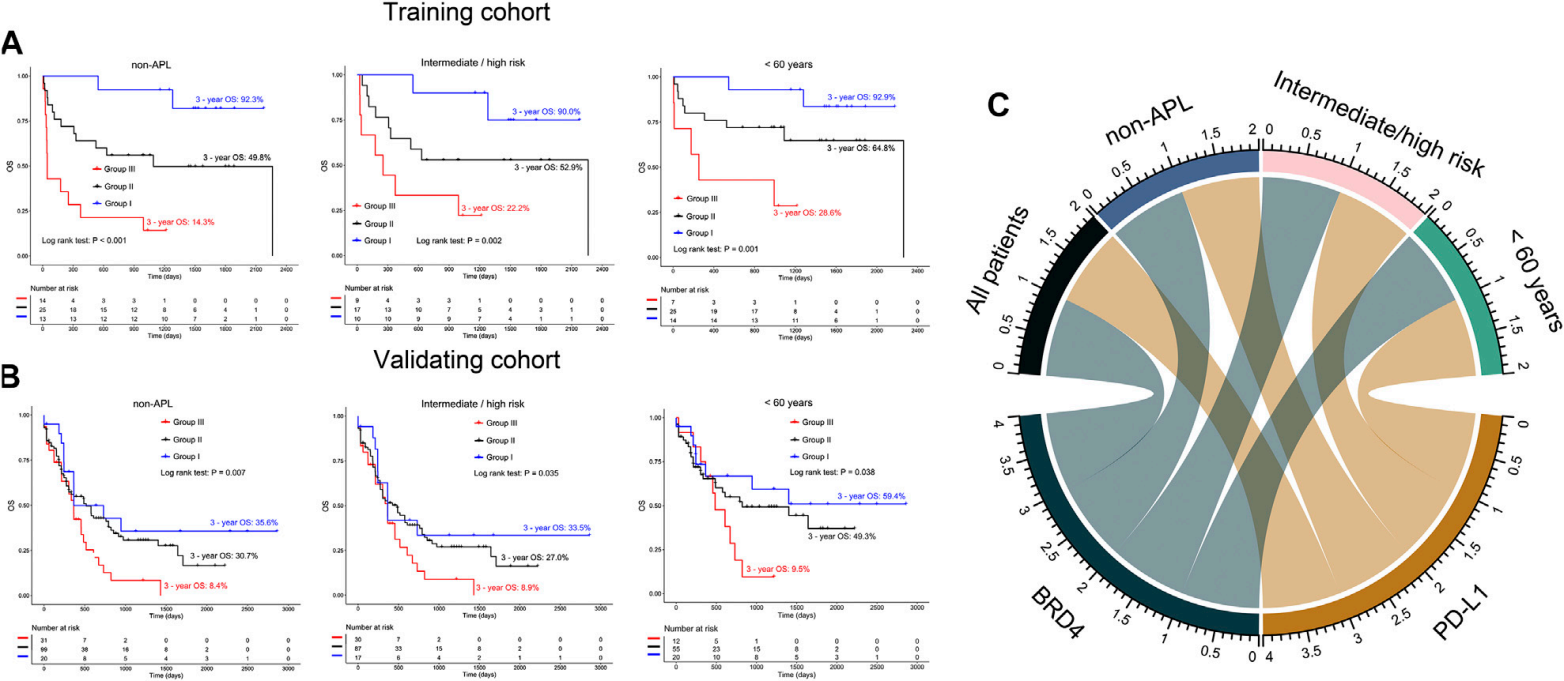
Subgroup analysis on co-expression of BRD4 and PD-L1 in predicting poor OS of AML patients. **(A,B)** Kaplan-Meier curves are shown for non-APL patients (left panel), intermediate/high risk (middle panel), and patients under 60 years old (right panel) according to the co-expression status of BRD4 and PD-L1 in the training **(A)** and validating **(B)** cohorts. **(C)** Summary chart of co-expression of BRD4 and PD-L1 for predicting OS in subgroups of AML patients. Group I: BRD4^low^PD-L1^low^; Group II: BRD4^high^PD-L1^low^ or BRD4^low^PD-L1^high^; Group III: BRD4^high^PD-L1^high^.

**FIGURE 6:**
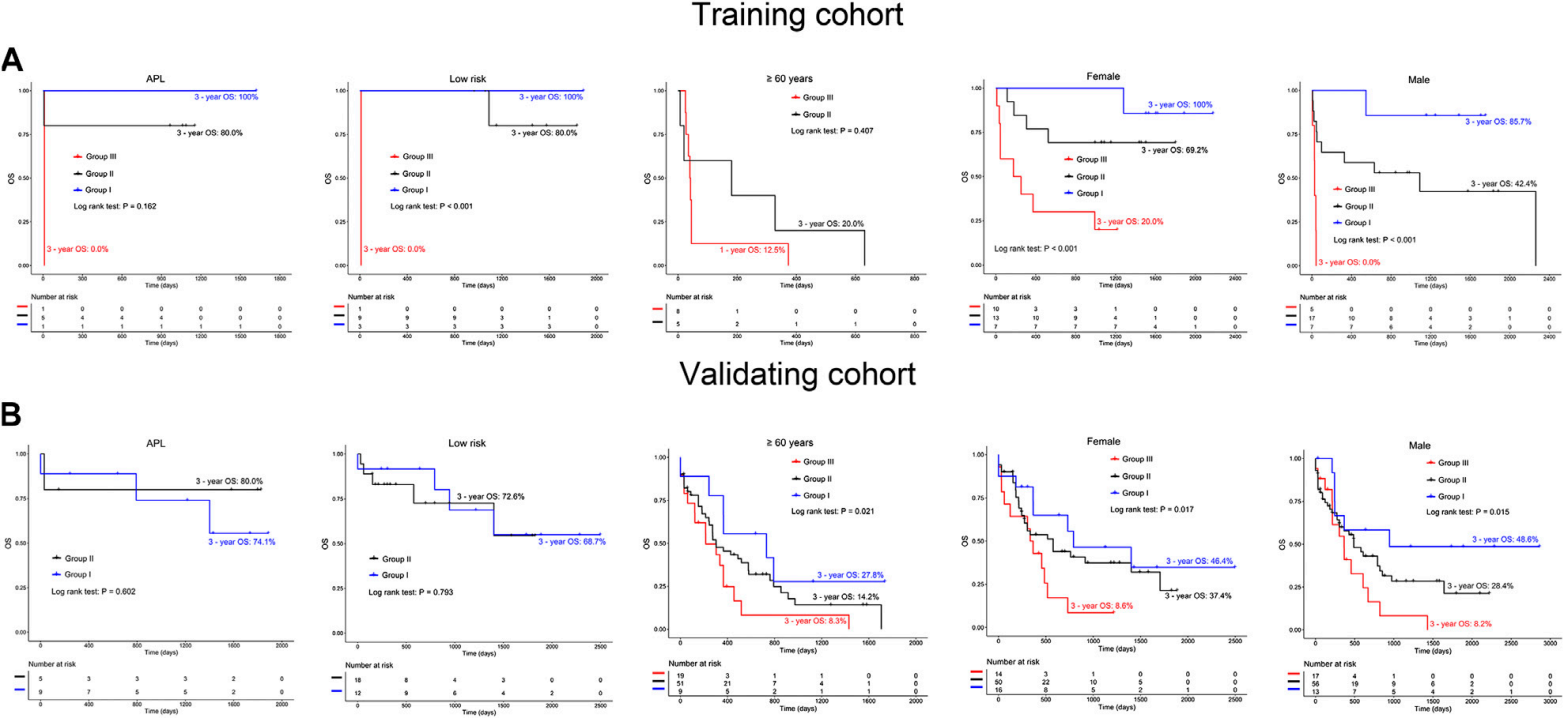
Comparison of OS in AML patients and those with either APL, low risk, ≥60 years old, females or males based on the expression of BRD4 and PD-L1 in the training **(A)** and validating **(B)** cohorts. Group I: BRD4^low^PD-L1^low^; Group II: BRD4^high^PD-L1^low^orBRD4^low^PD-L1^high^; Group III: BRD4^high^PD-L1^high^.

To explore the role of BRD4 and PD-L1 co-expression in predicting OS in specific groups of AML patients, we conducted subgroup analysis. In the training cohort, non-acute promyelocytic leukemia (non-APL) AML patients had longer OS in groups II and I (3 years: 49.8% vs. 92.3%), and shorter OS (3 years: 14.3%) in group III (*p* < 0.001). This finding was confirmed in the validating cohort (3-year OS: 8.4% vs. 30.7% vs. 35.6%, *p* = 0.007) (Figures 5A,B, left panel). Additionally, in the training and validating cohorts, group III appeared to have poor OS (3 years: 22.2 and 8.9%, respectively) compared to groups II and I (3 years: 52.9% vs. 90%, 27% vs. 33.5%, respectively) for AML patients with intermediate/high risk (Figures 5A,B, middle panel). Similarly, when BRD4 and PD-L1 co-expression was used to predict the OS of AML patients under 60 years old, a shorter OS was observed in group III compared to groups II and I in both the training (3 years: 28.6% vs. 64.8% vs. 92.9%, *p* = 0.001) and validating cohorts (3 years: 9.5% vs. 49.3% vs. 59.4%, *p* = 0.038) (Figures 5A,B, right panel). Co-expression of BRD4 and PD-L1 was associated with poor OS in both female and male patients with AML (*p* < 0.05, Figures 6A,B). However, co-expression of BRD4 and PD-L1 was unable to predict the OS of APL patients with low risk, or patients ≥60 years old (*p* > 0.05, Figure 6A,B).

## Discussion

BRD4 plays an important role in the progression and prognosis of various tumors, such as oral squamous cell carcinoma, ovarian cancer, and lymphoma (Zhu et al., 2016; Hogg et al., 2017; Wang et al., 2019). However, little is known about the prognostic value of BRD4 in patients with AML (Lu et al., 2017). In this study, we explored the association between the expression of BRD4 and the OS of AML patients. We found that over expression of BRD4 predicts poor OS in AML. This result supported the use of BRD4 inhibitors for chemotherapy in AML patients. However, ongoing clinical trials using BRD4 blockades in AML patients indicated that the efficacy of BRD4 single-agent blockade is low and drug resistance may occur (Braun et al., 2017). Therefore, new therapeutic strategies for combining other targeted drugs with BRD4 blockade are needed. Recently, studies have shown that combining ICB therapies with other targeted drugs have the potential to improve the anti-tumor response in AML (Daver et al., 2018; Stahl et al., 2019). Moreover, studies suggest that PD-L1 is a direct target of BRD4-mediated gene expression (Hogg et al., 2017). In this study, we found that the expression of BRD4 was positively correlated with PD-1 and PD-L1. Importantly, we found that co-expression of BRD4 and PD-L1 could better predict the OS of patients with AML than the co-expression of BRD4 and PD-1. This finding was consistent with our results in which knocking down BRD4 in AML cell lines inhibited PD-L1 expression (unpublished data). Moreover, subgroup analysis illustrated that co-expression of BRD4 and PD-L1 predicts poor OS in non-APL patients, patients under 60 years old, and those with intermediate/high risk. These findings may provide precise and valuable predictor of OS for AML.

Increasing evidence suggests that cancer patients with high TMB respond better to immunotherapy. Therefore, TMB is an important factor in evaluating the efficacy of ICB (Hellmann et al., 2018; Zhu et al., 2019). Notably, similar to high TMB, PD-L1 expression can enhance the clinical response of cancer patients who are treated with PD-1/PD-L1 inhibitors (Yarchoan et al., 2019). In this study, we found that AML patients with high TMB have poor OS, and that TMB was positively correlated with co-expression of BRD4 and PD-L1. Thus, combination of BRD4 and PD-L1 blockades might benefit AML patients who co-express BRD4 and PD-L1 and possess high TMB. However, a larger sample size is warranted for high-throughput sequencing to further validate the relationship among BRD4, PD-L1, and TMB in the future.

In summary, we demonstrate that transcriptome-based co-expression of BRD4 and PD-L1 could be a predictor of poor OS in AML patients, particularly in non-APL patients with intermediate/high risk or under 60 years old (Figure 4C). Furthermore, transcriptome-based co-expression of BRD4 and PD-L1 was positively correlated with TMB in AML. This finding will provide novel insights into designing combinational immuno-targeted therapy in AML patients.

## Data Availability Statement

The raw data supporting the conclusions of this article will be made available by the authors, without undue reservation, to any qualified researcher.

## Author Contributions

YL Contributed to the concept development, study design, and edited the manuscript. CC Collected the clinical information, interpreted the data, and wrote the manuscript. LX and RG Performed the experiments. CZ Contributed to the supervision of the experimental process and helped write the manuscript. CW, SW, and YZ Diagnosed and treated the patients and provided clinical bone marrow samples. All authors read and approved the final manuscript.

## Funding

This work was supported by grants from the National Natural Science Foundation of China (Nos. 82070152, 81770152, 91642111, and 81500126), the Guangzhou Science and Technology Project (Nos. 201807010004, 201803040017, and 201904010033).

## Conflict of Interest

The authors declare that the research was conducted in the absence of any commercial or financial relationships that could be construed as a potential conflict of interest.
