# Supplementary figures and images for: Transcriptome-Based Co-Expression of BRD4 and PD-1/PD-L1 Predicts Poor Overall Survival in Patients With Acute Myeloid Leukemia
Source: Front Pharmacol. 2021 Feb 1;11:582955. doi: 10.3389/fphar.2020.582955 (PMC7917577; doi:10.3389/fphar.2020.582955)

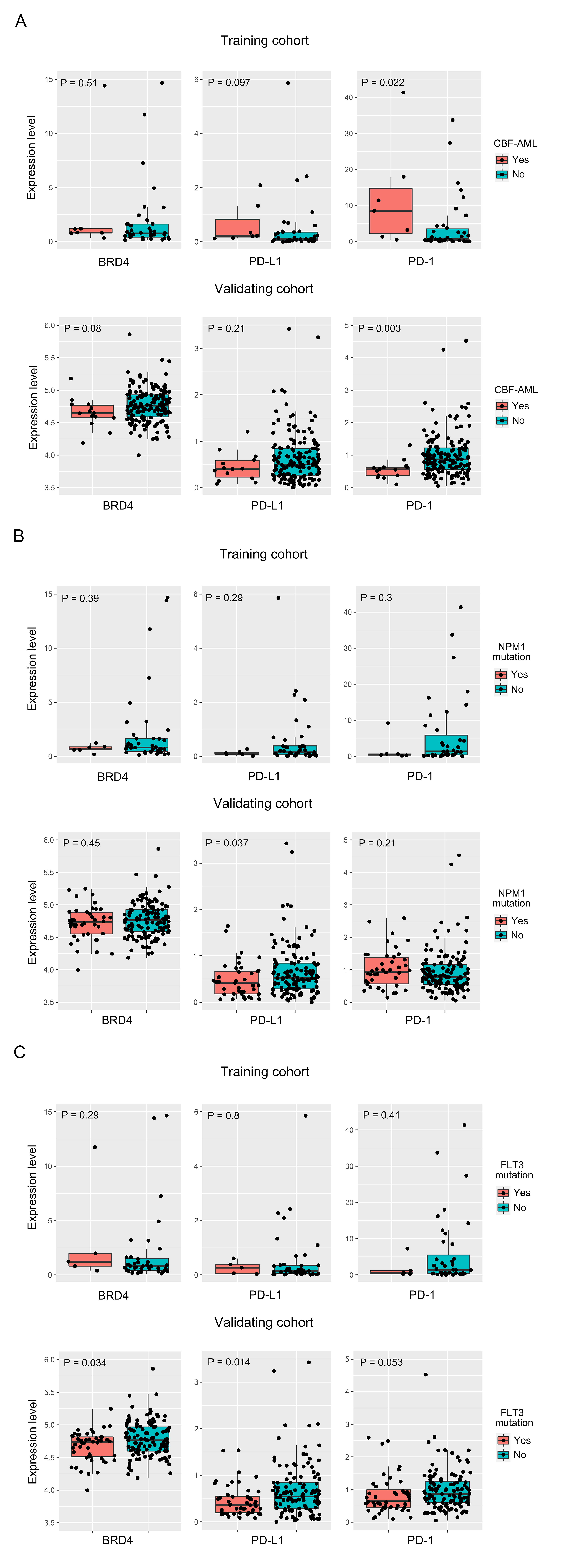

Supplement: Supplementary file 1 [file image1.tif]
